# A generic deviance detection principle for cortical On/Off responses, omission response, and mismatch negativity

**DOI:** 10.1007/s00422-019-00804-x

**Published:** 2019-08-19

**Authors:** Vincent S. C. Chien, Burkhard Maess, Thomas R. Knösche

**Affiliations:** Max Planck Institute for Human Cognitive and Brain Sciences, Stephanstraße 1a, Leipzig, Germany

**Keywords:** Deviance detection, Neural mass model, Auditory perception, Adaptation, NMDA

## Abstract

Neural responses to sudden changes can be observed in many parts of the sensory pathways at different organizational levels. For example, deviants that violate regularity at various levels of abstraction can be observed as simple On/Off responses of individual neurons or as cumulative responses of neural populations. The cortical deviance-related responses supporting different functionalities (e.g., gap detection, chunking, etc.) seem unlikely to arise from different function-specific neural circuits, given the relatively uniform and self-similar wiring patterns across cortical areas and spatial scales. Additionally, reciprocal wiring patterns (with heterogeneous combinations of excitatory and inhibitory connections) in the cortex naturally speak in favor of a generic deviance detection principle. Based on this concept, we propose a network model consisting of reciprocally coupled neural masses as a blueprint of a universal change detector. Simulation examples reproduce properties of cortical deviance-related responses including the On/Off responses, the omitted-stimulus response (OSR), and the mismatch negativity (MMN). We propose that the emergence of change detectors relies on the involvement of disinhibition. An analysis of network connection settings further suggests a supportive effect of synaptic adaptation and a destructive effect of *N*-methyl-d-aspartate receptor (NMDA-r) antagonists on change detection. We conclude that the nature of cortical reciprocal wiring gives rise to a whole range of local change detectors supporting the notion of a generic deviance detection principle. Several testable predictions are provided based on the network model. Notably, we predict that the NMDA-r antagonists would generally dampen the cortical Off response, the cortical OSR, and the MMN.

## Introduction

Automatic detection of sudden acoustic changes crucially enables reorientation of attention toward relevant events in the environment and thereby is important for survival. From a functional perspective, sensitivity to stimulus deviation likely plays many roles in the nervous system (e.g., noise rejection, duration tuning, chunking and grouping, beat perception, see reviews in [64, 111]) and enriches the hierarchical representations of percepts. The ability to detect abrupt temporal changes is thought to be a pervasive property of the sensory systems, given that deviance-related responses have been widely observed from cellular to system levels, across species, sensory modalities, and spanning from the lower levels of the sensory pathway to the cortex. For example, some cells can be sensitive only to the onsets and offsets of stimuli. These On/Off responses have been observed using extracellular recording in the superior paraolivary nucleus (SPON) of rodents [9, 23, 25, 48, 51], inferior colliculus (IC) of chinchillas [28], and the medial geniculate body (MGB) of the guinea pig [30]. Cortical On/Off responses have been observed using different recording and imaging techniques, including single-cell recording in primary auditory cortex (A1) of awake cats [18, 75], and anesthetized rats [86], extracellular recording in A1 of awake marmoset monkeys [82], surface micro-electrode array in auditory cortex (AC) of rats [97], multi-unit extracellular recordings across broad range of AC of mice [43], flavoprotein fluorescence imaging [4] and two-photon calcium imaging [4, 24] in AC of mice, and MEG in human auditory evoked responses [67]. Generally speaking, these cells can be sensitive to the sudden changes in specific regular features such as the constancy in pitch, loudness, duration, and patterns. The deviants that violate these perceptual regularities trigger mismatch responses at different stages such as frequency following responses (FFR), middle latency responses (MLR), as well as long latency responses (LLR) such as the mismatch negativity (MMN) [91]. An omitted stimulus in a periodic train of stimuli is a special type of deviant, which elicits the so-called omitted-stimulus responses/potentials (OSRs/OSPs). The OSR is time-locked not to the last but to the omitted stimulus, which reflects temporal expectancy represented in the neural circuits. OSRs have been observed in different sensory systems (e.g., visual, auditory, somatosensory) in various species, for example, the visual pathway of fish, reptile, and invertebrate in vivo [14, 44, 74, 77], retinas of salamander in vitro [89, 109], and the electrosensory system of rays [16]. An OSR at the cortical level (often termed the omission response or omission MMN) has also been observed in human EEG/MEG [2, 15, 17, 33, 45]. To date, investigations of the underlying mechanisms have been mostly confined to a certain perceptual level and a particular phenomenon. A unifying view of deviance detection that considers phenomena across levels is still missing.

Many of the deviance-related activities, though originating from different stages of the auditory pathway, can be observed pervasively in the auditory cortex. We hypothesize that the cortical deviance-related activities are primarily generated locally through reciprocally connected neural circuits. In this study, we outline a *generic deviance detection* principle, in an effort to reconcile some confusion and conflict related to the questions as follows.

**Which neural circuits give rise to the diverse cortical On/Off responses?** The response of a neuron or a neural circuit to a prolonged stimulus can bear three basic features: a response to the stimulus onset (On response), a sustained response as long the stimulus is present, and a response to the stimulus offset (Off response). The On/Off responses are found in neurons of the superior paraolivary nucleus (SPON) of the brainstem, the inferior colliculus (IC) of the midbrain [26], and the auditory cortex in rodents [4, 24, 86]. These On/Off neurons are thought to support functions such as duration selectivity (duration tuning), gap detection, and noise rejection [111]. Knowledge of the generation of On/Off responses has been mainly derived from observations at non-cortical stages. The On responses are thought to be due to adaptive and post-onset inhibitory mechanisms that shape the responses in the auditory nerve [72]. The Off responses are widely accepted to arise from post-inhibitory rebound (see review in [47] for the detailed cellular and synaptic mechanisms), as concluded from observation in SPON neurons [25]. Other response patterns such as On-Off, On-sustained-Off can then potentially be explained by mixing of excitatory and inhibitory inputs with different delays in a feed-forward network [111]. As for the On/Off responses recorded in the auditory cortex, they may originate from the ascending non-cortical On/Off responses [86] or be generated locally in the cortex. The cortical On/Off neurons show diverse temporal profiles [24]. Also, a single cortical neuron may have distinct onset- and offset-frequency receptive fields (FRFs) [75]. It is still unclear how the neural circuits give rise to these properties of cortical On/Off responses.

**Is the OSR just sustained resonance?** The OSR, elicited by an unexpected omission in periodic stimuli, is found in the cortex [2, 15, 17, 33, 45], but not in the midbrain (IC, tectum) [67] or the brainstem [55], where only Off responses are observed. The OSR resembles the Off response as they both peak at the end of a stimulus (or a train of stimuli) However, the OSR also reflects temporal expectancy (i.e., neural representation of periodicity), which distinguishes it from the Off response. There are two properties of the OSR. First, the peak latency includes an additional constant delay (e.g., around 100 ms in human MEG/EEG) from the time when the missing stimulus would have occurred (due time). It does not depend on the stimulus-onset asynchrony (SOA) [2, 90]. Second, the peak amplitude can be larger than the entrained responses during periodic stimuli [33]. Although neural activities that show sustained resonance can be a mechanism underlying the temporal expectancy [57, 99], sustained response alone does not explain the additional delay and higher peak amplitude. How the neural circuits maintain the input periodicity and detect the change is unclear.

**Does the OSR reflect prediction or prediction error?** This question rests on whether the OSR is triggered by a similar mechanism as the MMN. The MMN, elicited by a deviant among repetitive standard stimuli, is a negative deflection in the event-related potential (ERP) with the sources most prominently localized in the auditory cortex. The underlying process leads to the reorientation of attention to higher cognitive processes. MMNs have been shown for auditory deviants involving pitch [37, 62, 69, 70, 84, 98, 100, 113, 114], intensity [63, 79], duration [1, 19, 34–36, 40, 63, 65, 81, 87, 105], SOA [13, 50, 102], sequence (or pattern) [12, 32, 49, 88, 101, 117], and more complex features such as rising and falling tones (reviewed in [71]) or voice [46]. The MMN is generally accepted to be elicited by the deviant that violates the regularities, but the underlying mechanism is still under debate. The MMN is thought to reflect either a prediction-error signal resulting from the comparison between the input and the top-down prediction (prediction hypothesis), or an increased signal caused by the stimulus propagating through un-adapted synapses (adaptation hypothesis). The omission paradigms that elicit the OSR are often used in the debate to emphasize the need for active prediction, since the adaptation mechanism alone does not produce extra neural activities without any input. However, according to the computational models based on either hypothesis, the OSR is qualitatively different from the classical MMNs elicited by other deviants. The adaptation-based model suggests the OSR to be a rebound response (i.e., sustained resonance) rather than a modulated N1 [57]. The prediction-based model suggests the OSR to reflect predictive signals rather than prediction error [108]. Both interpretations implicitly suggest pure endogenous activities that do not involve a change detection mechanism. This conflicts with the two properties of ORS mentioned above. How OSR relates to MMN generation is not yet clear.

**Fig. 1:**
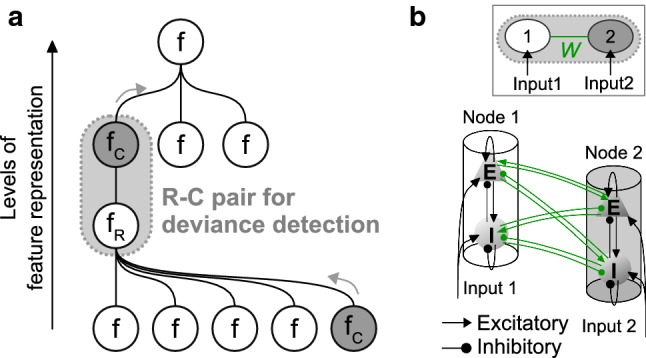
Illustration of the role of deviance detection in hierarchical feature representation. **a** The process of feature representation includes the interaction between regularity formation (R) and change detection (C). The R nodes remain stable (regularity, $f_\mathrm{R}$) by accumulating the ascending information from the lower-level features. The C nodes detect abrupt temporal changes in the neighboring R node(s) and pass them to the higher levels as new features ($f_\mathrm{C}$, gray arrows). In this sense, an R–C pair forms a basic mechanism of deviance detection which takes place at every level in the hierarchy. **b** An R–C pair is formed by two reciprocally coupled nodes. In the simulations, all nodes are allowed to receive external weighted inputs that reach the excitatory and inhibitory populations. The inter-node connections (green) are the free parameters, and the intra-node connections are fixed for simplicity (colour figure online)

The above issues underscore the need for a unifying view of deviance detection, covering the cortical On/Off responses, the cortical OSR, and the MMN. Given the relatively uniform wiring patterns across areas in the cortex, we ask whether cortical deviance detection is supported by neural circuits of a common structural motif. We propose a *generic deviance detection* principle (Fig. 1a), where change detection can take place locally under proper reciprocal connections (Fig. 1b) by monitoring the neighboring neural activities that represent a regular feature. This principle is based on the assumption that the process of deviance detection can be functionally separated into stages of *regularity formation* and *change detection*.

In the first part of the Results section, we provide simulation examples that reproduce several properties of cortical On/Off responses, cortical OSR, and MMN. In examples I and II, we demonstrate that the various types of cortical On/Off responses, in terms of their temporal profiles and frequency receptive fields (RFRs), can be attributed to the connection patterns between input and observation points. In example III, we demonstrate that the OSR can be regarded as a change detection response (or an Off response) to the cessation of constant periodicity. In example IV, we demonstrate that the sequence MMN can be regarded as a change response to the switch in sequence regularity (or a mixture of an On response to the deviant and an Off response to the cessation of regularities). In the second part of the Results section, we examine the underlying mechanism of change detection by investigating the generation of simulated On and Off responses. We then look at how altered connection patterns (e.g., reduced external connections to inhibitory populations, effect of NMDA-r antagonists, and synaptic adaptation) affect the emergence of change detectors. In the Discussion section, we derive conclusions with regard to the above-mentioned questions. Finally, we provide testable predictions for future verification.

## Methods

### Model description

The simulations are done with rate-based models which allow for a simple and scalable network motif while keeping the network dynamics comparable to the experimental observations such as LFP and MEG/EEG. A network is used to represent an area in the auditory cortex with each node in the network comprising one excitatory (*E*) and one inhibitory (*I*) neural population. The dynamics of the *E* and *I* populations are represented by the overall post-synaptic membrane potential (PSP) $v^p(t)$ and the mean firing rate $m^p(t)$, where the superscript $p\in \{E,I\}$ stands for the excitatory/inhibitory population. Neural populations interact with each other by means of firing rate via connections defined in the matrices $W^{EE}$, $W^{IE}$, $W^{EI}$ and $W^{II}$ which correspond to excitatory-to-excitatory, excitatory-to-inhibitory, inhibitory-to-excitatory, and inhibitory-to-inhibitory connections, respectively. Self-feedback is allowed. All *E* populations in the network are fed with constant background input. External stimuli *x*(*t*) reach the *E* and *I* populations via external connections specified by $W^{EX}$ and $W^{IX}$.

#### Neural populations

In neural mass modeling, the processing of neural activities in a population is governed by two operators [38, 39, 93, 94]. The *rate-to-potential operator* describes a linear transformation from the mean firing rate to the mean PSP. The input firing rate $x_c(t)$, where the subscript $c\in \{e,i\}$ stands for the excitatory/inhibitory synapse, reaches a population and is transformed to the EPSP/IPSP $v_c(t)$ in that population. This transformation is described by the rate-to-potential process, which is achieved by convolving the input firing rate $x_c(t)$ with a synaptic kernel $h_c(t)$.

1 $$\begin{aligned} v_c(t)=x_c(t) \otimes h_c(t) \end{aligned}$$

The synaptic kernel $h_c(t)$ is a response curve describing the dynamics of the post-synaptic potential in response to a pre-synaptic spike, which depends on the characteristics of the synapse (Eq. 2). The average synaptic gain $H_c$ controls the peak value of the response curve. The time constant $\tau _c$ represents the delay due to dendritic effects and neurotransmitter kinetics. The symbol $\varTheta (t)$ denotes the Heaviside step function, where $\varTheta (t\ge 0)=1$.

2 $$\begin{aligned} h_c(t)=\frac{H_c}{\tau _c}t\varTheta (t)e^\frac{-t}{\tau _c} \end{aligned}$$

The convolution of the input $x_c(t)$ with the kernel $h_c(t)$ can be further represented by two first-order ordinary differential equations (Eqs. 3,4), which are used in the numerical simulation:

3 $$\begin{aligned} \dot{v_c}(t)= & {} u_c(t) \end{aligned}$$

4 $$\begin{aligned} \dot{u_c}(t)= & {} \frac{H_c}{\tau _c}x_c(t)-\frac{2}{\tau _c}u_c(t)-\frac{1}{\tau _c^2}v_c(t) \end{aligned}$$

Finally, $v_c(t)$, representing either the EPSP $v_e(t)$ or the IPSP $v_i(t)$, contributes to the overall PSP *v*(*t*) in the neural population.

5 $$\begin{aligned} v(t)=v_e(t)-v_i(t) \end{aligned}$$

The *potential-to-rate operator* transforms the overall PSP *v*(*t*) into the output firing rate *m*(*t*) by a nonlinear sigmoid function *S* as described in Eq. 6, where $e_0$ controls the maximum firing rate and *r* controls the slope at the membrane potential $v_0$ for firing.

6 $$\begin{aligned} m(t)=S\left( v(t) \right) =\frac{2e_0}{1+e^{r(v_0-v(t))}} \end{aligned}$$

#### Nodes

A node, consisting of one excitatory and one inhibitory neural population, represents the basic building block in a hierarchical feature representation (Fig. 1). It represents more of a functional unit than a structural unit, for example a cortical column. For *N* nodes that represent *N* locations in the auditory cortex and *M* external inputs that represent the intensity of a certain feature such as *M* tones, the network structure is defined by four $N \times N$ connection matrices $W^{EE}$, $W^{IE}$, $W^{EI}$ and $W^{II}$, and two $N \times M$ external connection matrices $W^{EX}$ and $W^{IX}$. Each element *w* (non-negative) in the connection matrices stands for the gain factor on the firing rate, which reflects the number and strengths of the synapses established from the source to the target population. The element $w_{jk}^{EI}$, for example, stands for the connection strength from the inhibitory population in node *k* to the excitatory population in node *j*. The overall PSP *v*(*t*) of the excitatory population in node *j* is now labeled as $v_j^E(t)$ and is composed of the respective EPSP $v_{j,e}^{E}(t)$ and IPSP $v_{j,i}^{E}(t)$:

7 $$\begin{aligned} v_j^E(t)=v_{j,e}^{E}(t)-v_{j,i}^{E}(t) \end{aligned}$$

$v_{j,e}^{E}(t)$ and $v_{j,i}^{E}(t)$ are found by solving the differential equations:

8 $$\begin{aligned}&{\dot{v}}_{j,e}^{E}(t)=u_{j,e}^{E}(t)\end{aligned}$$

9 $$\begin{aligned}&{\dot{u}}_{j,e}^{E}(t) = \frac{H_e}{\tau _e}\left[ \sum _{k=1}^N a_{jk}w_{jk}^{EE}m_k^E(t)+\sum _{q=1}^M w_{jq}^{EX}x_q(t)+B \right] \nonumber \\&\qquad \qquad \quad -\frac{2}{\tau _e}u_{j,e}^{E}(t)-\frac{1}{\tau _e^2}v_{j,e}^{E}(t) \end{aligned}$$

10 $$\begin{aligned}&{\dot{v}}_{j,i}^{E}(t)=u_{j,i}^{E}(t)\end{aligned}$$

11 $$\begin{aligned}&{\dot{u}}_{j,i}^{E}(t)=\frac{H_i}{\tau _i}\left[ \sum _{k=1}^N w_{jk}^{EI}m_k^I(t) \right] -\frac{2}{\tau _i}u_{j,i}^{E}(t)-\frac{1}{\tau _i^2}v_{j,i}^{E}(t)\nonumber \\ \end{aligned}$$

In Eqs. 9 and 11, $w_{jk}^{EE}$, $w_{jk}^{EI}$, and $w_{jq}^{EX}$ are elements in $W^{EE}$, $W^{EI}$, and $W^{EX}$. The $m_k^E(t)$ and $m_k^I(t)$ are the firing rate of the excitatory and inhibitory population in node *k*. The $x_q(t)$ is external input *q*, and *B* is a constant background input. The synaptic adaptation term $a_{jk}$ modulates the connections strength $w_{jk}^{EE}$.

Similarly, the overall PSP *v*(*t*) of the inhibitory population in node *j* is labeled as $v_j^I(t)$ and is composed of the respective EPSP $v_{j,e}^{I}(t)$ and IPSP $v_{j,i}^{I}(t)$:

12 $$\begin{aligned} v_j^I(t)=v_{j,e}^{I}(t)-v_{j,i}^{I}(t) \end{aligned}$$

$v_{j,e}^{I}(t)$ and $v_{j,i}^{I}(t)$ are found by solving the differential equations:

13 $$\begin{aligned}&{\dot{v}}_{j,e}^{I}(t)=u_{j,e}^{I}(t) \end{aligned}$$

14 $$\begin{aligned}&\begin{aligned} {\dot{u}}_{j,e}^{I}(t)=&\frac{H_e}{\tau _e}\left[ \sum _{k=1}^N w_{jk}^{IE}m_k^E(t)+\sum _{q=1}^Mw_{jq}^{IX}x_q(t) \right] \\&-\frac{2}{\tau _e}u_{j,e}^{I}(t)-\frac{1}{\tau _e^2}v_{j,e}^{I}(t) \end{aligned} \end{aligned}$$

15 $$\begin{aligned}&{\dot{v}}_{j,i}^{I}(t)=u_{j,i}^{I}(t) \end{aligned}$$

16 $$\begin{aligned}&{\dot{u}}_{j,i}^{I}(t)=\frac{H_i}{\tau _i}\left[ \sum _{k=1}^N w_{jk}^{II}m_k^I(t) \right] -\frac{2}{\tau _i}u_{j,i}^{I}(t)-\frac{1}{\tau _i^2}v_{j,i}^{I}(t)\nonumber \\ \end{aligned}$$

The value of constant background input *B* is chosen such that the nodes work in proper conditions (i.e., near a bifurcation point for an isolated node). The external input $x_q(t)$ reaches both the excitatory and inhibitory populations in node *j* with connection strengths $w_{jq}^{EX}$ and $w_{jq}^{IX}$, where the ratio $w_{jq}^{IX}/w_{jq}^{EX}$ is set to 0.5 by default. The synaptic adaptation term *a* represents the efficacy of excitatory-to-excitatory connections $W^{EE}$. The synaptic efficacy (in range [0,1]) varies according to Equation 17 when synaptic adaptation is considered, otherwise *a* is fixed to 1.

#### Synaptic adaptation

When synaptic adaptation on $W^{EE}$ is considered, the connection strength $w_{jk}^{EE}$ is modulated (as in Eq. 9) by the term $a_{jk}$, which varies according to the pre-synaptic activity $m_k^E(t)$.

17 $$\begin{aligned} {\dot{a}}_{jk}(t)=\frac{1-a_{jk}(t)}{\tau _a}-\kappa a_{jk}(t)m_k^E(t) \end{aligned}$$

The adaptation time constant $\tau _a$ represents the recovery rate of the synaptic efficacy, and the constant $\kappa$ influences the decay rate of $a_{jk}(t)$.

#### Short-term plasticity

Short-term plasticity is used only in simulation example III as a possible solution for the regularity formation of input periodicity. The plasticity rule adjusts the binding between the nodes in the bank of oscillators so that the group activity maintains a stable representation of input periodicity. For $N_b$ nodes in the bank of oscillators, the connection $w_{jk}^{EE}$ increases if the covariance $Cov_{j,k,\varDelta t}(t)$ between $m_j^E(t)$ and $m_k^E(t)$ from time $t-\varDelta t$ to *t* is positive, and otherwise decreases gradually back to zero (Eq. 18). Similarly, the connection $w_{jk}^{EI}$ increases if $Cov_{j,k,\varDelta t}(t)$ is negative, and otherwise decreases gradually back to zero (Eq. 19). The learning rate $\eta$ is set to 0.05, and the weight masks $\alpha _{jk}$ and $\beta _{jk}$ consider the effectiveness of plasticity as a function of the distance between nodes *j* and *k*. The weight masks follow the Gaussian function ${\hbox {exp}}(-d^2/2\sigma ^2)$, where $d=|j-k|$, and $\sigma$ is set to $0.2N_b$ and $0.4N_b$ for $\alpha _{jk}$ and $\beta _{jk}$, respectively. Since the resonance frequency increases monotonically with the node index in the bank of oscillators, the weight masks avoid the binding between two nodes with distinct resonance frequencies. So far, this short-term plasticity rule is rather function-driven than based on biological evidence. The plasticity rule is not the focus of this study because we assume that short-term plasticity is more involved in the process of regularity formation than in change detection. More studies need to be done for a more realistic network model that maintains the input periodicity.

18 $$\begin{aligned} {\dot{w}}_{jk}^{EE}(t)= & {} -w_{jk}^{EE}(t)+\eta \alpha _{jk} \cdot \hbox {max}\big (Cov_{j,k,\varDelta t}(t),0 \big ) \end{aligned}$$

19 $$\begin{aligned} {\dot{w}}_{jk}^{EI}(t)= & {} -w_{jk}^{EI}(t)+\eta \beta _{jk} \cdot |\hbox {min}\big (Cov_{j,k,\varDelta t}(t),0 \big )| \end{aligned}$$

#### Simulated MEG signals

To synthesize a gross signal from the activities of all neural populations in the network, both the excitatory current (or active sink) and inhibitory current (or active source) at the excitatory populations (i.e., pyramidal cells) are taken into account [22]. This is a more generalized approach than just considering the sum of the excitatory inputs weighted by excitatory-to-excitatory connection strength and the adaptation term [59]. For the network of *N* nodes, the simulated MEG signal *R*(*t*) is calculated as the weighted sum of currents contributed by the active sinks and sources. It is assumed that the active sinks are due to the EPSP at apical dendrites through $W^{EE}$, and the active sources to the IPSP at the soma through $W^{EI}$. In order to highlight the activities of specific nodes (e.g., the change detectors), the signals are weighted by *b*, where $\sum _j^Nb_j=1$.

20 $$\begin{aligned} R(t)=\sum _{j=1}^N b_j \left[ \sum _{k=1}^N a_{jk}(t)w_{jk}^{EE}m_k^E(t)+ \sum _{k=1}^Nw_{jk}^{EI}m_k^I(t) \right] \nonumber \\ \end{aligned}$$

**Table 1:** General configurations

Par.	Value	Unit	Description
*Impulse response function*			
$\tau _e$	10	ms	Time constant of average delay through excitatory synapses
$\tau _i$	20	ms	Time constant of average delay through inhibitory synapses
$H_e$	3.25	mV	Average gain through excitatory synapses
$H_i$	22	mV	Average gain through inhibitory synapses
*Sigmoid function*			
$e_0$	2.5	spikes/s	Controlling maximum firing rate
*r*	0.56	1/mV	Slop at $v_0$
$v_0$	6	mV	Membrane potential threshold for firing
*Intra-node connections*			
$w_{jj}^{EE}$	$135\times 0.8$	1	Self-feedback excitatory synapses in the *j*th excitatory population
$w_{jj}^{IE}$	$135\times 0.6$	1	Excitatory synapses from the *j*th excitatory to the *j*th inhibitory population
$w_{jj}^{EI}$	$135\times 0.2$	1	Inhibitory synapses from the *j*th inhibitory to the *j*th excitatory population
$w_{jj}^{II}$	$135\times 0.05$	1	Self-feedback inhibitory synapses in the *j*th inhibitory population
*Inter-node connections*			
$w_{jk}^{EE}$	$135\times \{0,0.1,\ldots ,0.5\}$	1	Excitatory synapses from the *k*th excitatory population to the *j*th excitatory population
$w_{jk}^{IE}$	$135\times \{0,0.1,\ldots ,0.5\}$	1	Excitatory synapses from the *k*th excitatory population to the *j*th inhibitory population
$w_{jk}^{EI}$	$135\times \{0,0.1,0.2\}$	1	Inhibitory synapses from the *k*th inhibitory population to the *j*th excitatory population
$w_{jk}^{II}$	$135\times \{0,0.1,0.2\}$	1	Inhibitory synapses from the *k*th inhibitory population to the *j*th inhibitory population
*External connections*			
$w_{jq}^{EX}$	$220\times 0.2$	1	Excitatory synapses from external input *q* to the *j*th excitatory population
$w_{jq}^{IX}$	$220\times 0.1$	1	Excitatory synapses from external input *q* to the *j*th inhibitory population
*Inputs*			
*B*	$220\times 2.5$	spikes/s	Constant background input
$x_q(t)$	–	spikes/s	External input *q* (step function, amplitude$\,=\,$1.5, rise/fall time$\,=\,$10 ms)
*Synaptic adaptation*			
$\tau _a$	200	ms	Time constant of recovery rate of synaptic efficacy
$\kappa$	2	1	Drop rate in synaptic efficacy

### Model configurations

The parameter settings of neural population model are kept the same, as proposed by Jansen and Rit [38] and Jansen et al. [39], unless otherwise specified. In order to reduce the number of free parameters, we fix the intra-node connections and only analyze the inter-node connections in the simulations. The values of intra-node connections are chosen such that a single node stays inactivated under weak excitatory input and starts to oscillate as the excitatory input strength increases to $e_0$ (i.e., half of the maximum value of the sigmoid function). The adaptation parameters $\tau _a$ and $\kappa$ are chosen such that a single node remains oscillating during prolonged stimulation, rather than showing only a transient peak response at the onset. The general configurations are listed in Table 1.

### Categorization of network behavior

**Fig. 2:**
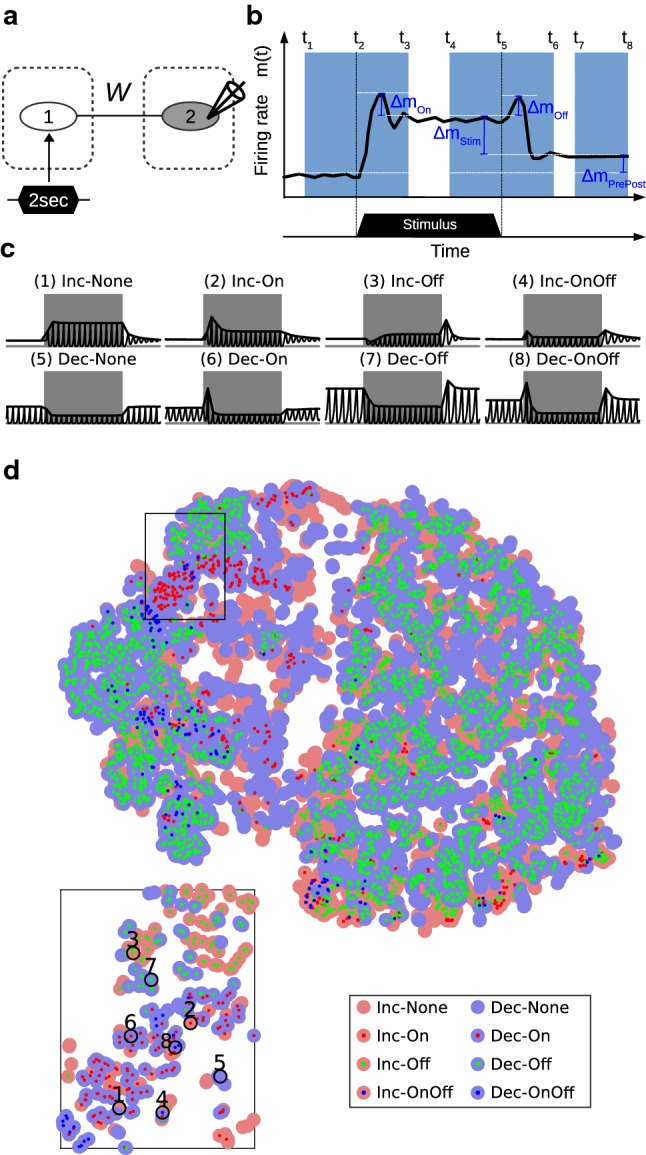
Change detectors and the corresponding *W* solutions. **a** In the simulation settings, a prolonged stimulus of 2000 ms is fed to node 1. A range of inter-node connections *W* are scanned through, and the various temporal behaviors of the change detector (i.e., time courses of $m_2^E(t)$) are categorized. **b** For categorization, four variables $\varDelta m_{On}$, $\varDelta m_{Stim}$, $\varDelta m_{Off}$, and $\varDelta m_{PrePost}$ are calculated according to the time windows (light blue areas) for each time course $m_2^E(t)$. The time courses that are not bistable are then categorized as one of the eight On/Off types. See detailed categorization settings in Table 2. **c** The exemplary responses of eight On/Off types. Gray bands represent the duration of stimulus. The black curves represent the time courses of $m_2^E(t)$, and the bold black curves are the envelopes. **d** All *W* solutions of the eight On/Off types in the scanned range are projected onto a 2D plane for visualization (MATLAB function: tsne), where color dots represent the eight types ($\{$Inc, Dec$\}\times \{$None, On, Off, OnOff$\}$). The eight exemplary behaviors in **c** are labeled in the zoomed in area (colour figure online)

In a two-node network where a prolonged stimulus (2000 ms) is fed to node 1 (Fig. 2a), the behavior of node 2 (i.e., the time course $m_2^E(t)$) is categorized as one of the nine types based on the level changes and the peak at edges: (1) Inc-None, (2) Inc-On, (3) Inc-Off, (4) Inc-OnOff, (5) Dec-None, (6) Dec-On, (7) Dec-Off, (8) Dec-OnOff, and (9) others. (See Fig. 2b and Table 2 for details of categorization.) The ‘Inc’ and ‘Dec’ stand for increased and decreased activities during the stimulus. The ‘On,’ ‘Off,’ and ‘OnOff’ stand for transient peak(s) only at the onset, the offset, or both, of the stimulus. ‘None’ stands for no clear peaks at the edges of the stimulus. Bistable or non-responsive behaviors are categorized as others.

In Fig. 2c, we show eight exemplary On/Off types. Note that the envelope drawn in Fig. 2c is mainly for visualization and to demonstrate the various transient behaviors. From the viewpoint of simulation, the network does not always oscillate. The oscillation happens under a certain range of connection patterns among *E*/*I* populations. The tendency to oscillate depends a lot on the intra-node connection settings. In our simulation, the Off response relies on the interaction among *E*/*I* populations, but does not necessarily depend on the oscillating behavior. From the viewpoint of experimental observation, there are evoked oscillations during/after the stimulus in the thalamo-cortical auditory system [20].

## Results

**Fig. 3:**
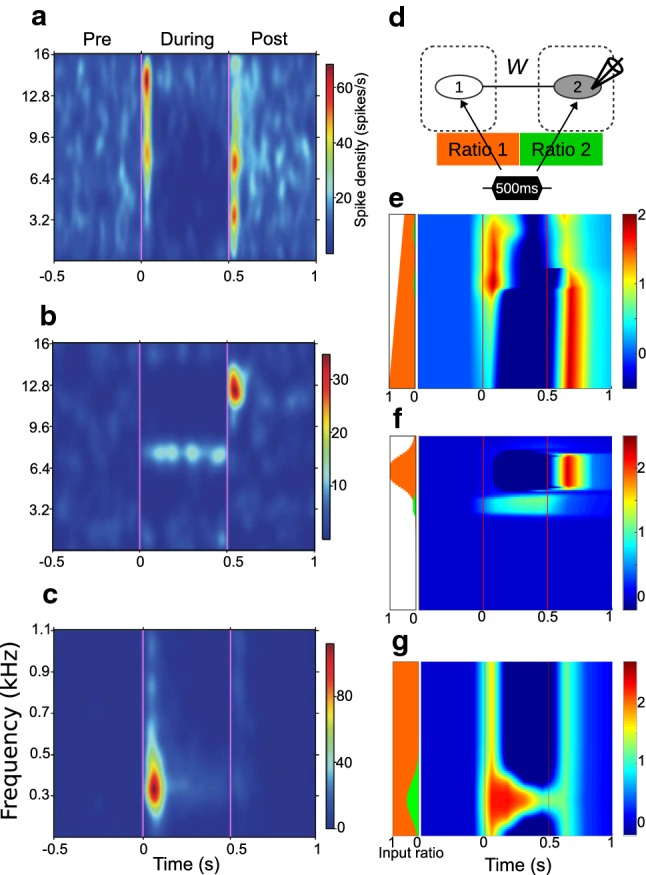
Distinct onset and offset FRFs. **a–c** Three exemplary cells that show distinct onset and offset FRFs (adapted from [75]). The cells were recorded in the primary auditory cortex in awake cats. Sound stimuli of pure tone (ranging from 128 to 16,000 Hz) were presented for 500 ms. The pre-, during-, and post-stimulus spike densities of the cell are color coded. **d** In the simulation settings, a two-node network with adjustable external connections $w_1^{EX}$ and $w_2^{EX}$ (orange and green color) is used to mimic the experimental observations. **e–g** Simulation results that mimic the observations in **a–c**. The green and orange input ratios at the left-side bar of each plot represent the settings of external connections $w_1^{EX}$ and $w_2^{EX}$ for each simulation trial. The firing rate is color coded. The pre-stimulus firing rate is used as baseline, and the negative value (deep blue color) during the stimulus represents decreased activity (colour figure online)

**Fig. 4:**
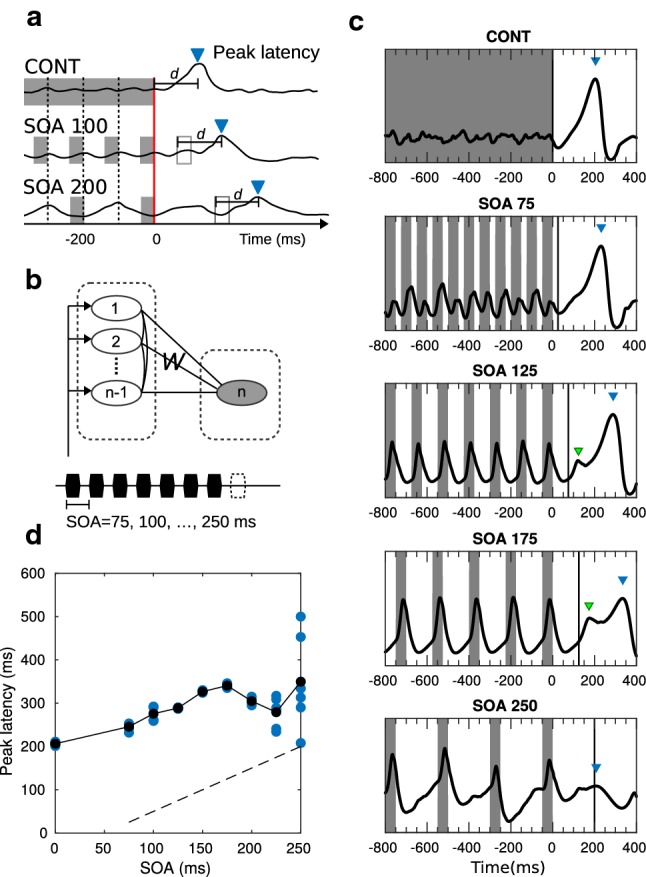
Omitted-stimulus response (OSR). **a** Illustrative responses that show temporal expectation. The peak latencies should be linear to the SOA if the offsets of stimuli are aligned (red line), or a constant d if the due times are aligned (empty rectangles). See, for example, Figure 2 in [2]. **b** In the simulation settings, the R nodes (left column) are simply implemented with different time constants $\tau _e$ and $\tau _i$, leading to different resonance frequencies. A prolonged stimulus or periodic stimuli are fed to these R nodes. **c** The simulated MEG signals (black curves) rise after the due time (black vertical lines) and show different peak latencies and peak amplitudes (marked with blue triangles). The small peaks (marked with green triangles) reflect the momentum of the bank of oscillators. **d** Simulated peak latencies are linear to the SOA. Simulations are run for several trials for each SOA where the offset time is changed. The peak latencies in each simulation trial (blue dots) under the same SOA can be different, which depends on the network stability during the stimulus and the offset time. Black dots are the mean peak latencies. The peak latencies show an approximately constant delay with respect to due time (time of predictable omission, dashed line) when the SOAs are below 200 ms. The peak latencies become unstable across trials when SOAs are above 200 ms. In other words, the temporal expectation is preserved in this network for SOAs smaller than 200 ms (colour figure online)

**Fig. 5:**
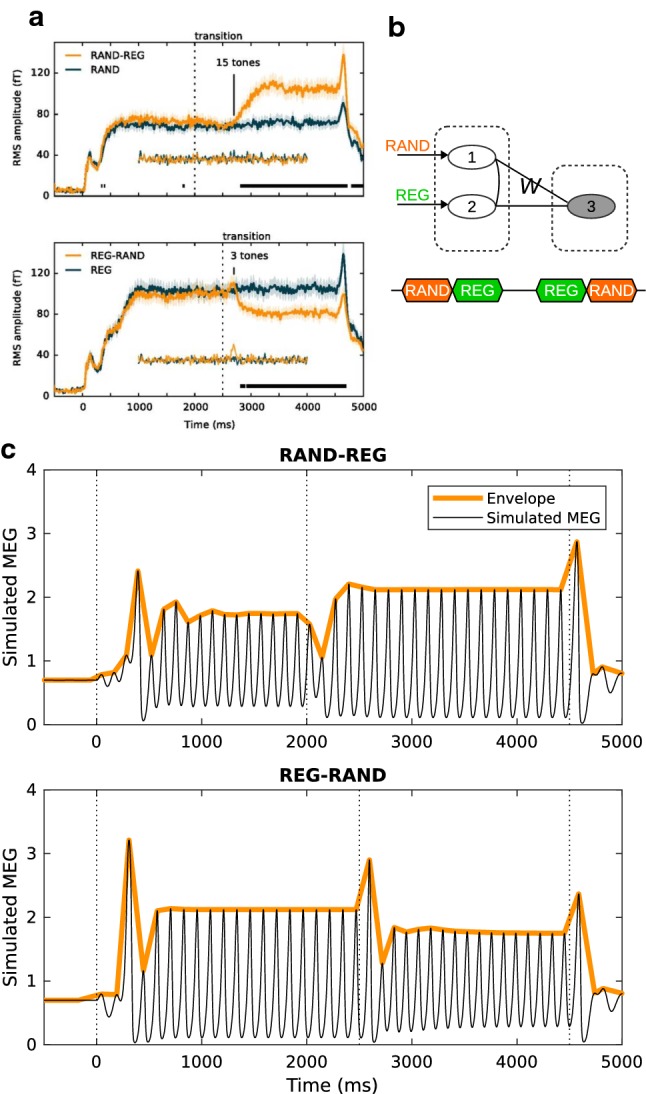
Sequence MMN (roving paradigm). **a** Brain responses to transitions between regular and random sound sequences (adapted from [5]). There are transient peaks at the onsets/offsets of sound sequences as well as at the transitions from regular (REG) to random (RAND) sequences. In addition, the RMS amplitude is higher during regular sequences. **b** In the simulation settings, a three-node network is used for mimicking the observation in **a**. The R nodes (nodes 1 and 2) receive the stimulus inputs representing RAND and REG, respectively. The inter-node connections *W*, between the C node (node 3) and the R nodes, are picked up from the *W* solutions such that the C node shows Inc-OnOff responses to the stimulus inputs. The connections between the R are tuned to result in the different level shifts during the stimulus and the elimination of the transient peak at the transition from RAND to REG. **c** Simulated MEG signals of the three-node network

**Fig. 6:**
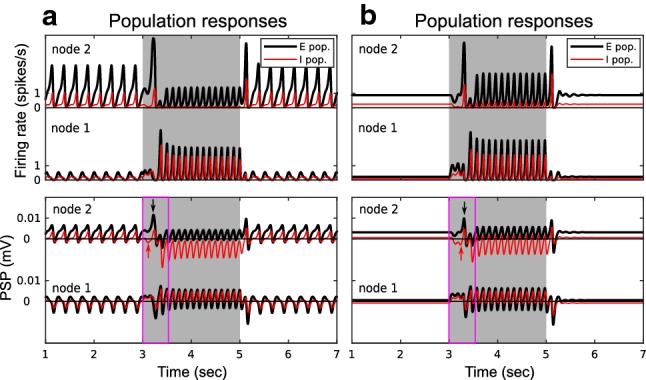
The generation of On responses. **a** In the simulation settings, a prolonged stimulus of 2000 ms is fed to the R node (node 1) in a two-node network. The inter-node connections *W* is chosen from the *W* solutions that give rise to Dec-OnOff responses in the C node (node 2) (see Fig. 2b). The node responses to the stimulus (gray period) are shown as firing rates (upper plot) and as PSPs (lower plot). As an Dec-OnOff response, the time course $m_2^E(t)$ (black curve) shows a lower amplitude during the stimulus and transient peaks at the onset and offset of the stimulus. To understand the generation of On response in population $E_2$, the magenta rectangle indicates the period of transient disinhibition where population $I_2$ is transiently inhibited by population $I_1$ (red arrow), and population $E_2$ peaks right after the transient disinhibition (black arrow). **b** A similar example for Inc-OnOff response, where the generation of an On response is also due to the transient disinhibition (colour figure online)

**Fig. 7:**
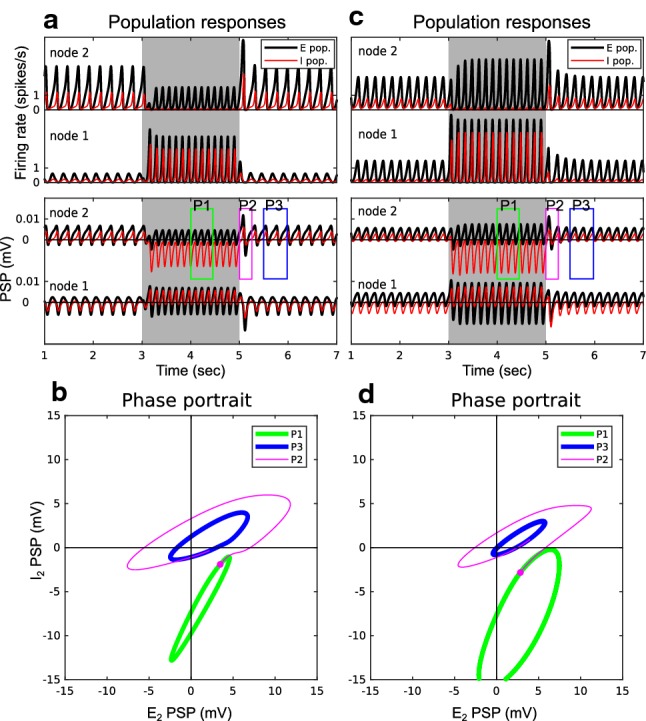
The generation of the Off responses. **a** In the simulation settings, a prolonged stimulus of 2000 ms is fed to the R node (node 1) in a two-node network. The inter-node connections *W* is chosen from the *W* solutions that give rise to Dec-Off responses in the C node (node 2) (see Fig. 2b). The node responses to the stimulus (the gray period) are shown as firing rates (upper plot) and as PSPs (lower plot). As an Dec-Off response, the time course $$m_2^E(t)$$ (black curve) shows a lower amplitude during the stimulus and a transient peak at the offset of stimulus. The population $$I_2$$ is strongly inhibited during the stimulus, which is reflected by the negative PSP of population $$I_2$$$$v_2^I(t)$$ (red curve in the green rectangle). The disinhibition is followed by the Off response in population $$E_2$$ thereafter (black curve in the magenta rectangle). **b** Phase portraits (*P*1: during stimulus, *P*2: offset of stimulus, *P*3: post-stimulus) of node 2. The phase portrait *P*3 (i.e., when there is only background input) runs counter-clockwise, and the phase portrait *P*1 (i.e., during the stimulus) shifts downward and runs clockwise, reflecting the strong inhibition of $$I_2$$. The phase portrait *P*2 shows the transient trajectory of transition from *P*1 to *P*3. The magenta dot denotes the time of stimulus offset. **c** The simulation settings for a Inc-Off response. The firing rate $$m_2^E(t)$$ shows higher amplitude during the stimulus and a transient peak at the offset of the stimulus. Same as in **a**, population $$I_2$$ is strongly inhibited during the stimulus, which is then followed by the Off response. **d** The phase portraits are similar to **b** except that the amplitude of $$v_2^E(t)$$ is larger during *P*1 than *P*3. The two examples show that the generation of Off responses is not relevant to the increased or decreased activities in $$E_2$$ , but to the inhibition on $$I_2$$ during the stimulus (colour figure online)

**Fig. 8:**
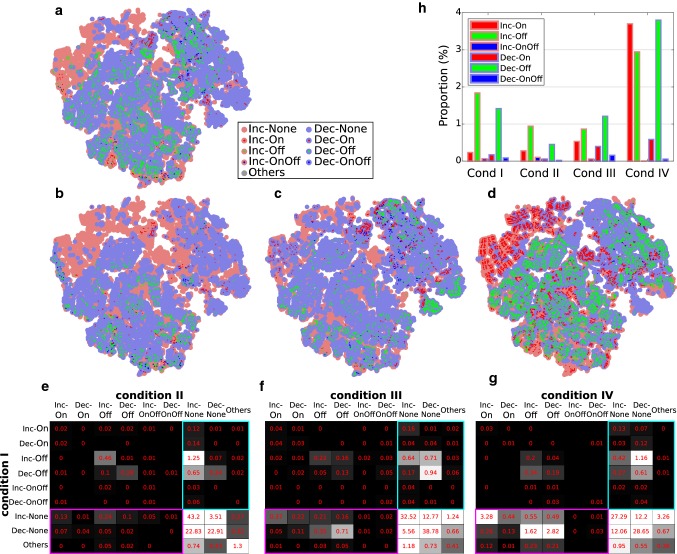
The effects of factors influencing the strength of connections *W* on the occurrence of On/Off responses. The *W* solutions of On/Off responses projected onto a 2D plane under **a** condition I: default, **b** condition II: $W^{IX}=0$, **c** condition III: NMDA-r antagonist, and **d** condition IV: synaptic adaptation. Dots with different colors and sizes represent different response types. **e** The contingency table of *W* solutions for condition I versus II. The value in each cell of the table (in red, with grayscale background) is the number of *W* solutions over the total number of scanned *W*s. A cell without a value means there was no *W* solution in that case. The cyan and magenta rectangles highlight the *W* solutions of On/Off types under one condition but not under the other. **f** Condition I versus III. **g** Condition I versus IV. **h** The bar chart represents the proportions of *W* solutions of On/Off types under the four conditions (colour figure online)

**Fig. 9:**
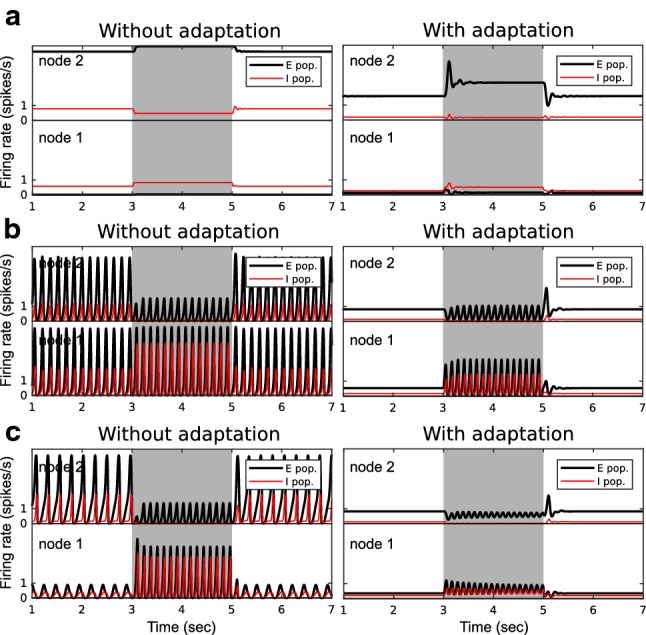
Network responses without and with synaptic adaptation. **a** Example of Inc-None type response in population $E_2$ under condition I (i.e., no adaptation) turning into Inc-On type under condition IV (i.e., when synaptic adaptation is applied on $W^{EE}$). **b** Example of Dec-None type turning into Inc-Off type. **c** Example of Dec-None type turning into Dec-Off type

The generic deviance detection principle suggests that deviance detections take place locally in the perceptual hierarchy as illustrated in Fig. 1. Any two reciprocally coupled nodes in a network can potentially form an R–C pair that serves deviance detection. The connections within an R–C pair can be heterogeneous across locations, thus giving rise to various behaviors of change detectors. In what follows, we reproduce some observed phenomena of deviance-related responses using simple networks (e.g., comprising two, three, and twenty one nodes) in simulation examples (Sect. 3.1), and then, we investigate the behavior of change detectors and the corresponding network settings in Sect. 3.2. The MATLAB code for Figs. 2, 3, 4, 5, 6, 7, 8 and 9 is found in Github: http://github.com/vscChien/gddp.

**Table 2:** Settings and variables for categorization of network behavior

Par.	Value	Unit	Description
*Time windows*			
$[t_\mathrm{On},t_\mathrm{Off}]$	[0, 2000]	ms	Stimulus onset and offset
$[t_1,t_2]$	$[-500,0]$	ms	Pre-onset window
$[t_2,t_3]$	[0, 500]	ms	Post-onset window
$[t_4,t_5]$	[1500, 2000]	ms	Pre-offset window
$[t_5,t_6]$	[2000, 2500]	ms	Post-offset window 1
$[t_7,t_8]$	[3500, 4000]	ms	Post-offset window 2
*Variables comparing*$max\left( m^E(t_i<t<t_j)\right)$			
$\varDelta m_\mathrm{PrePost}$	–	spikes/s	Bistability check ($[t_1,t_2]$ versus $[t_7,t_8]$)
$\varDelta m_\mathrm{Stim}$	–	spikes/s	Level change ($[t_4,t_5]$ versus $[t_1,t_2]$ and $[t_7,t_8]$)
$\varDelta m_\mathrm{On}$	–	spikes/s	Onset peak height ($[t_1,t_2]$ versus $[t_2,t_3]$)
$\varDelta m_\mathrm{Off}$	–	spikes/s	Offset peak height ($[t_4,t_5]$ versus $[t_5,t_6]$)
*Thresholds*			
$\theta _\mathrm{PrePost}$	0.1	spikes/s	**Bistable** behavior, if $\varDelta m_\mathrm{PrePost}\ge \theta _\mathrm{PrePost}$
$\theta _\mathrm{Stim}$	0	spikes/s	**Increased** response, if $\varDelta m_\mathrm{Stim}>\theta _\mathrm{Stim}$
			**Decreased** response, otherwise
$\theta _\mathrm{On}$	0.5	spikes/s	**On** response, if $\varDelta m_\mathrm{On}>\theta _\mathrm{On}$
$\theta _\mathrm{Off}$	0.5	spikes/s	**Off** response, if $\varDelta m_\mathrm{Off}>\theta _\mathrm{Off}$

### Simulation examples

#### Example I: temporal profiles of cortical On/Off responses

A prolonged tone stimulus can elicit diverse temporal patterns of On/Off responses in the auditory cortex. Neurons can be sensitive to the onset/offset of the stimulus (i.e., transient responses at the edges) and also show increased or decreased firing rate during the stimulus (i.e., level changes) compared with the spontaneous activity [18, 24, 43, 75, 78, 106]. In this simulation, we fed the input stimulus (2000 ms duration) to a two-node network (Fig. 2a), where the change detector does not directly receive the input stimulus (i.e., the external connections to node 2, $w_2^{EX}=w_2^{IX}=0$). Varying the inter-node connections *W* alters the response of the change detector (e.g., the firing rate of its excitatory population $m_2^E(t)$). We scanned a range of inter-node connections ($W^{EE},W^{IE}\in \{0,0.1,\ldots ,0.5\}$; $W^{EI},W^{II}\in \{0,0.1,0.2\}$), and categorized each of the time courses of $m_2^E(t)$ as one of the eight types, based on the level changes and the peak at edges (Fig. 2b). The *W* solutions are connection settings that give rise to one of the eight categorized On/Off types under these specific simulation settings (e.g., the intensity and onset/offset time of stimulus, the intensity of background input, and intra-node connections, etc).

To further investigate the relation between the inter-node connections *W* and the On/Off responses, we projected the *W* solutions $\{W_{type\_i},i=1,2,\ldots ,8 \}$ onto a 2D plane by t-Distributed Stochastic Neighbor Embedding [56]. This allowed the visualization of the mutual proximity of *W* solutions in the original eight-dimensional space. We expected to see clear clusters of different On/Off types, but the result was not always like that. This means that a certain On/Off type cannot be simply attributed to certain types of connection. Instead, the On/Off type is very sensitive to the inter-node connections *W*. From Fig. 2d, we observe several things. (1) Although the *W* solutions exhibit a clustered pattern, from a broad perspective, different types are observed when zooming in. The clustering patterns and their sensitivity to *W* may potentially explain the diverse, but spatially clustered On/Off responses shown in Figure 5 of [24]. (2) The Off types are not constrained within Inc/Dec clusters, suggesting that Off responses are not crucially determined by the level change of $m_2^E(t)$ during the stimulus. (3) The On and Off types occupy distinct areas in the 2D plane, which agrees with the conclusion that On and Off responses are driven by largely nonoverlapping sets of synaptic inputs [86]. (4) However, there are also areas where the On, Off and OnOff types are close to each other, where neuroplasticity (e.g., synaptic adaptation, spike-timing-dependent plasticity, or homeostatic plasticity) may play a role in changing the neural response from one type to another.

#### Example II: distinct onset- and offset-frequency receptive fields (FRFs)

As demonstrated in Example I, the two-node network can account for the different temporal profiles of On/Off responses. A network with the same properties can account for the distinct onset and offset FRFs in individual cells in the auditory cortex [75]. For example, the exemplary cell in Fig. 3a is sensitive to the onsets of sound stimuli at higher frequencies (3200–15,872 Hz) and the offsets of sound stimuli at lower frequencies (512–16,000 Hz), as reflected by higher spike density (yellow and red). In addition, this cell shows suppressed spike density (deep blue) during stimuli at low and middle frequencies. In short, the On/Off responses vary across tonal frequencies and across cells.

In the simulation, we used the two-node network to reproduce the distinct FRFs in Fig. 3a–c. For each simulation trial, the stimulus input (500 ms duration), corresponding to a pure tone in one trial of the experimental recordings, was fed to both nodes with different external connection strength (i.e., $w_1^{EX}=44\times ratio1$; $w_1^{IX}=22\times ratio1$; $w_2^{EX}=44\times ratio2$; $w_2^{IX}=22\times ratio2$, as in Fig. 3d). In Fig. 3d, the ratios (orange and green) reflect how far the two nodes are from the stimulus source. Considering the tonotopic organization in the auditory cortex, the ratios were also changed for each simulation trial because the stimulus input in each trial represented a different tonal frequency. The inter-node connections *W* were picked up from the *W* solutions, were fixed in each example, and the ratios adjusted such that the responses of node 2 (i.e., the time courses of $m_2^E(t)$ qualitatively mimicked the experimental observations. The simulation trials were then merged to make simulated FRFs (Fig. 3e–g).

In Fig. 3e, the excitatory population $E_2$ shows a Dec-Off response when $ratio1=1$ and $ratio2=0$ (the same as the ideal case used in Example I). The On response emerges as ratio1 decreases, and a small amount of *ratio*2 results in stronger On responses and weaker Off responses. In Fig. 3f, $E_2$ shows a Dec-Off response when $ratio1=1$ and $ratio2=0$, and turns into Inc-None type when *ratio*2 is larger than *ratio*1. In Fig. 3g, $E_2$ shows a Dec-OnOff response with the *ratio*2 values associated with the On responses.

The two-node network, although rate-based, may provide a sense of how the exemplary cells in Fig. 3a–c are influenced by different sound tones: *ratio*2 (green) indicates which tones are closer to (or more directly influencing) the cell, whereas *ratio*1 (orange) reflects how its surrounding neurons are sensitive to the tonal scope.

#### Example III: omitted-stimulus response (OSR)

The OSR resembles the Off response as they both peak at the offset of a prolonged stimulus or a train of periodic stimuli. However, the OSR is differentiated from the Off response by its property of temporal expectation. The peak latencies of OSR are not constant but proportional to the stimulus-onset asynchrony (SOA) of the repetitive stimuli as illustrated in Fig. 4a. The OSR at the cortical level (i.e., omission response or omission MMN) resembles the classic MMN, as both responses are related to violations to certain expectations (e.g., expectation of ‘when’ or ‘what’ concerning the stimuli).

The generic deviance detection principle suggests that the cortical OSR is a change detection response (or an Off response) to the end of a stable periodicity representation. In our simulation, the periodicity was represented by a *bank of oscillators* [53, 57] comprising multiple nodes (i.e., R nodes) with different resonance frequencies (implemented by different time constants $\tau _e$ and $\tau _i$ for simplicity). The use of a bank of oscillators is based on the observation that the auditory cortex shows a spatial representation of both frequency and periodicity [7, 8, 52]. In the bank of oscillators, the temporal feature of periodicity is transformed into a spatial pattern represented by the R nodes. Unlike the conventional bank of oscillators, that are not connected to each other, the R nodes are inter-connected with short-term plasticity on $W^{EE}$ and $W^{EI}$. Note that applying short-term plasticity on $W^{EE}$ and $W^{IE}$ also works well in this example. The plasticity enhances the connections between two nodes if they oscillate with high covariance, while it reduces the connections otherwise (Eqs. 18 and 19). This enables the resonance among R nodes to be sustained after the due time. The change detector (C node) that connects to the R nodes (as in Fig. 4b) is expected to peak when the sustained resonance drops. In Fig. 4c, we simulated MEG signals resulting from prolonged (CONST) and periodic stimuli (SOAs: 75, 125, 175, and 250 ms; stimulus duration: 50 ms). The OSR peaks are marked by blue triangles. When the SOA was increased, the peak latency increased and the peak amplitude decreased, which is in line with MEG observations [2]. The small peak before the OSR (particularly clear for SOA 125 and 175) is located at the time of the omitted stimulus, which resembles the expected evoked potential before the OSR (e.g., Figure 7B in [14]). In Fig. 4d, we show that the n-node network ($n=21$ in this example) is able to respond with the correct timing (i.e., a constant delay after the detectable omission) if the SOA is within 150 ms. The peak latencies become unstable for SOAs larger than 200 ms. This limitation is due to the limit of resonance frequencies in the bank of oscillators. As shown in Fig. 4c, the simulated MEG data for SOA 250 is not as stable compared to the faster SOAs.

In this example, we have demonstrated that the cortical OSR can reflect a detection mechanism upon the stable representation of periodicity. The sustained resonance was crucial for temporal expectation. This is in line with the observation that the auditory brainstem does not generate overt OSRs [55], likely because sustained resonance has not happened at that stage. Source analysis, as well as fMRI, showed that the OSR (more specifically, the fast OSR [45]) is localized to the auditory cortex [2, 60, 76, 116], suggesting that the auditory cortex has the capacity to represent a certain range of periodicity locally (e.g., under 200 ms). However, we have not yet fully investigated the neural mechanism underlying temporal expectancy. The bank of oscillators, which only assumes heterogeneity across neural populations, is so far a good candidate for implementation.

#### Example IV: sequence mismatch negativity (MMN)

The responses in a roving paradigm reveal the progress of regularity formation and change detection, and thus are useful for demonstrating the generic deviance detection principle. In Fig. 5a, an MEG study shows how the human brain responds to the switch between regular and random complex acoustic patterns [5]. There are On and Off responses at the onsets and offsets of the stimulus sequence. An MMN response is elicited by the transition from regular to random sequences (REG-RAND), while there is only a gradually rising root mean square (RMS) amplitude the other way around (RAND-REG). Also, the RMS amplitude is higher during regular sequences compared to random sequences.

In the simulation, we used a three-node network to reproduce the temporal profile of the RMS in Fig. 5a. Two stimulus inputs (REG and RAND) that represent the random and regular features were fed to nodes 1 and 2, respectively, as in Fig. 5b. The intensity and rise/fall time of the two stimulus inputs were the same as in the previous examples, and the durations were set to match the experiment in [5]. The inter-node connections *W* between nodes 1,2 and node 3 were chosen from the *W* solutions in Fig. 2d. The connections between nodes 1 and 2 did not have to be symmetric and were manually tuned to match the observed RMS. In Fig. 5c, the simulated MEG signal shows (1) On and Off responses at the onset and offset of stimulus sequences, (2) MMN response to the transition from regular to random sequences (REG-RAND), and (3) different RMS amplitudes during REG and RAND presentations.

The three-node network demonstrates how the inter-node connections *W* among the three nodes alone can account for the transient responses to the onsets and offsets, the selectivity to the direction of transition, as well as the level changes in RMS amplitude during random or regular sequences. For more realistic settings, the rise/fall time of the two stimulus inputs can be set differently. For example, it is reasonable to set a longer rise time for the REG stimulus input because it takes some time (at least a sequence length) to form regularity representation. This also explains why there is no MMN in the RAND-REG transition. Moreover, the intensity of the two stimulus inputs may reasonably be set differently because the status of neural populations under regular and random sequences can be dramatically different, which explains the level changes in RMS amplitude. In this simulation example, we used identical stimulus inputs, in an attempt to highlight the effect of inter-node connections *W* on the shaping of the network activity. Note that this simulation example sheds light on the contribution of a change detector, rather than the details of regularity formation. To understand how the REG sequence causes higher RMS amplitude, we assumed short-term plasticity on $W^{EE}$ and $W^{IE}$ in the lower-level neural populations at the stage of regularity formation. This follows the suggestion by a dynamic causal modeling study [3] that synaptic gain modulation in the auditory cortex is involved in processing regular sequences.

### The requirements for a change detector

The generic deviance detection principle emphasizes the ubiquity of local change detection and its separation from regularity formation. In the previous simulation examples, we demonstrated that the behavior of a change detector can account for many phenomena (e.g., diverse cortical On/Off responses, distinct onset and offset FRFs, cortical OSR, and sequence MMN). Here, we present a more detailed analysis of the exact requirements for a change detector to work. First, we investigated how and under which conditions the On and Off responses occur. Then, we examined how changes in connection strengths affect the generation of On/Off responses through three factors: (1) external input to inhibitory populations, (2) blockage of NMDA receptor channels, and (3) synaptic adaptation.

#### The generation of On responses

It has been proposed that On responses could be due to adaptive and post-onset inhibitory mechanisms that reshape the onset response in auditory nerve fibers [72]. In our simulations, we found that the On responses can also be due to the transiently inhibited activity of the inhibitory population $I_2$ at the onset of a stimulus. As shown in Fig. 6, population $I_2$ is shortly inhibited by population $I_1$ , and the low $v_2^I(t)$ leads to a transient peak in $v_2^E(t)$ (indicated by the red and black arrows in the magenta rectangles). The system returns to stability soon after the $v_2^E(t)$ peak brings $v_2^I(t)$ up again. These On responses were due to *transient disinhibition*; therefore, the inter-node connection $W^{II}$ plays an important role in the generation of the On responses.

#### The generation of Off responses

It is widely accepted that Off responses followed by decreased activity (i.e., the Dec-Off responses) arise from post-inhibitory rebound that is related to the intrinsic conductance property of the neuronal membranes [48]. However, the generation of Off responses that follow increased activity (i.e., the Inc-Off responses) cannot be simply explained by the post-inhibition mechanism (see review in [47, 111]). Next, we examined under which conditions the Dec-Off and Inc-Off responses might arise at the network level.

In Fig. 7, the population $E_2$ shows Off responses for both cases: the decreased and increased activity during a stimulus. In the simulations, both Dec-Off and Inc-Off responses resulted from the same mechanism. As shown in Fig. 7a, c, the Off response came in two steps. First, the population $I_2$ received strong inhibition from population $I_1$ during the stimulus (reflected by the negative PSP $v_2^I(t)$ during $t=3000$ to 5000 ms). Second, the population $E_2$ activity peaked before $I_2$ recovered after stimulus offset (the transient peak $v_2^E(t)$ during $t=5000$ to 5100 ms). The occurrence of Off responses can also be represented by phase portraits as shown in Fig. 7b, d. The trajectories of the phase portraits show how $v_2^E(t)$ and $v_2^I(t)$ evolved interactively. When there was only background input, $E_2$ and $I_2$ oscillated in the normal steady state (the counter-clockwise blue trajectories) where $E_2$ excites $I_2$, and $I_2$ inhibits $E_2$. During stimulus presentation, $E_2$ and $I_2$ oscillated in a reversed steady state (the clockwise green trajectories) where $E_2$ had an additional inhibitory effect on $I_2$ through the pathway $E_2 \rightarrow I_1 \rightarrow I_2$, and $I_2$ had an additional disinhibitory effect on $E_2$ through the pathway $I_2 \rightarrow I_1 \rightarrow E_2$ , due to the involvement of active $I_1$ during stimulus. The Off responses are depicted by the magenta trajectories during the transition from the reversed steady state to the normal steady state.

The simulations provide clues for the underlying neural mechanisms. The inter-node connection $W^{II}$ is critical for a network to give rise to the Off responses because the inhibitory population $I_2$ first has to be inhibited (i.e., disinhibition). The inter-node connection $W^{EI}$ is important to maintain the network in the working state (e.g., the reversed steady state), otherwise the network gets ‘overheated’ during disinhibition. With these structural prerequisites, the excitatory population $E_2$ may show a transient Off response before the inhibitory population $I_2$ catches up again following stimulus offset.

The timing of stimulus offset (i.e., the initial point in the state space when the transition begins) and other parameters that alter the trajectories of the two steady states (such as the stimulus intensity, and the settings of $W^{EE}$ and $W^{IE}$) also affected the generation of Off responses, but these factors were not critical. Moreover, the decreased activity during the stimulus is not critical for the generation of the Off response at network level (cf., it is necessary in the post-inhibitory mechanism at cellular level). As shown in Fig. 7d, the amplitude of $v_2^E(t)$ during the stimulus (green trajectory) can be larger compared to no stimulus (blue trajectory).

#### Factors influencing the On/Off responses

We considered the effect of three factors with respect to the generation of On/Off responses: (1) external input to inhibitory populations, (2) blockage of NMDA receptor channels, and (3) synaptic adaptation. More specifically, we assessed how each of these three factors influences the distribution of *W* solutions in the two-node network.

Since disinhibition played an important role in the generation of both On and Off responses, as illustrated in the above simulations (Figs. 6, 7), we were interested in seeing the contribution of external input to the inhibitory population $I_1$ . In condition II, the external connection $W^{IX}$ was set to zero in comparison with the default setting $W^{IX}=0.5W^{EX}$ (condition I).

The NMDA-r antagonist MK-801 is found to reduce inhibition during stimulation and thus to reduce the Off responses [4]. NMDA-r antagonists are also known to reduce the amplitude of the MMN [61]. In condition III, we mimicked the effect of NMDA-r antagonists by reducing the connection strength of $W^{EE}$ by 25$\%$ and reducing $W^{IE}$ by 50$\%$. The difference in reduction applied to the two connections was based on the fact that excitatory synapses on inhibitory neurons are mainly covered by NMDA channels and therefore are more sensitive to NMDA-r antagonists than the excitatory synapses on excitatory neurons [80]. The setting of external connections remained the same as the default setting. Note that in principle, both conditions II and III may be due to NMDA-r antagonists, because they are based on decreased excitatory input to the inhibitory populations. So, if NMDA-r antagonists are indeed the cause of reduced connection strengths to inhibitory populations, the effect in condition II and III should occur simultaneously. Other effects caused by NMDA-r antagonists, such as the changes in NMDA currents, synaptic plasticity and synaptic time constants, were not included.

The phenomenon of synaptic adaptation is ubiquitous in the nervous system and has been suggested to be one of the mechanisms underlying deviance detection. Since we suggest that deviance-related responses can be interpreted as change detection responses to regularity representation, it is important to know whether synaptic adaptation promotes the emergence of change detectors. In condition IV, the synaptic adaptation is considered. The intra- and inter-node connections $W^{EE}$ were modulated by the synaptic efficacy term *a* as described in Eq. 17. Note that as described in Eq. 9, the external input via $W^{EX}$ to the excitatory populations is not affected by synaptic adaptation.

The responses of the two-node network with a range of inter-node connections *W*s (as in Example I in Sect. 3.1) were simulated, and each *W* was assigned to one of the nine types of responses (Also see Fig. 2b). Four conditions were tested: (I) the default condition, where synaptic adaptation was not applied, and $W^{IX}=0.5W^{EX}$, (II) $W^{IX}=0$, (III) $W^{EE}=0$ reduced by 25$\%$ and $W^{IE}=0$ reduced by 50$\%$, and (IV) synaptic adaptation applied. To visualize the results, the *W* solutions of types 1 to 9 were projected onto a 2D plane (Fig. 8a–d). The number of W solutions under the four condition is summarized in the contingency table (Fig. 8e–g) and the bar chart (Fig. 8h).

The bar chart (Fig. 8h) shows that the number of *W* solutions of Off types in condition II was reduced compared to condition I. Most of the Off types under condition I became None types under condition II (e.g., Inc-Off $\rightarrow$ Inc-None among 1.25$\%$ of the scanned *W*s. See the cyan rectangle in Fig. 8e). This suggests that the external connection $W^{IX}$ is supportive of the generation of Off responses, because the $I_1$-to-$I_2$ disinhibition was enhanced due to the external input via $W^{IX}$.

In condition III, the number of *W* solutions of Off types was reduced, but the number of *W* solutions of On types was slightly increased, compared to condition I (Fig. 8h). This is in line with experimental results showing that NMDA-r antagonists reduce Off responses but On responses are not affected [41, 104].

In condition IV, the number of *W* solutions of both On and Off types was greatly increased (Fig. 8h). Many of the None types under condition I turned into On and Off types under condition IV (e.g., 3.28$\%$: Inc-None $\rightarrow$ Inc-On; 1.62$\%$: Dec-None $\rightarrow$ Inc-Off; 2.82$\%$: Dec-None $\rightarrow$ Dec-Off. See the magenta rectangle in Fig. 8g). This suggests that synaptic adaptation greatly promotes the emergence of change detectors. To see how synaptic adaptation alters the network responses, Fig. 9a–c shows three examples of altered responses due to synaptic adaptation. The three examples show typical type transitions from condition I to condition IV.

## Discussion

In this paper, we propose a *generic deviance detection* principle based on the observation that many deviance-related cortical responses occur without clear evidence of functionally specific wiring patterns. The proposed mechanism suggests that reciprocal wiring in the cortex gives rise to the emergence of change detectors that respond to abrupt changes in regular features. With this notion, the deviance-related responses observed in the cortex such as cortical On/Off responses, the cortical OSR and the MMN can be regarded as responses of change detectors at different levels of abstraction.

The simulation examples demonstrate that the network responses can indeed resemble the properties of cortical On/Off responses (Figs. 2, 3), the cortical OSR (Fig. 4), as well as the MMN (Fig. 5). We then investigated the wiring patterns in the network that support the generation of On/Off responses (Figs. 6, 7). The results suggest that the inhibitory-to-inhibitory connections are important for both On and Off responses, which implies that these deviance-related responses are closely related to disinhibition. In the simulations that mimicked the effect of NMDA-r antagonists and synaptic adaptation, the results show that NMDA-r antagonists suppress the Off responses and mildly promote On responses, whereas synaptic adaptation generally boosts both On and Off responses (Figs. 8, 9). In what follows, we provide our viewpoints regarding the questions raised in the introduction. Some testable predictions raised by our model are presented at the end of the discussion.

*Different processes in regularity formation, but same mechanism in change detection.* The generic deviance detection principle suggests that *change detection* may rely on a common neural mechanism (i.e., the local reciprocal wiring), while *regularity formation* may, depending on the level of abstraction, require different brain resources and time to collect relevant information.

There are a number of dissimilarities among deviance-related responses, which, discussed as follows, are mainly due to differences in the process of regularity formation. We take the differences between cortical OSR and MMN as an example. In terms of the temporal window of integration (TWI), a pitch MMN can be elicited by traditional oddball paradigms even when the SOA is larger than 500 ms [6, 83], while the estimated length of TWI for cortical OSRs is much shorter (160–170 ms) [112]. In terms of attention, it has been suggested that fast and slow periodic sequences elicit cortical OSRs by two different mechanisms: The fast OSR (periodicity > 5 Hz) is elicited automatically, while the slow OSR (periodicity < 2 Hz) requires the involvement of attention [45]. The slow OSR can be elicited at large SOAs such as 800 ms in [29]; and 1000 to 2000 ms in [17]. The need for attention suggests that the cortical OSR and MMN are different processes [66]. In terms of required repetition, a successful elicitation of MMN needs only two or three repetitions for simple feature-repetition regularities [10, 21, 96, 110], while the cortical OSR requires up to 9 repetitions in a train for a successful elicitation [33]. The above observations suggest different processes, related to the degree of difficulty in regularity formation, underlie cortical OSR and MMN.

There are also several similarities among the deviance-related responses that support the notion of a common mechanism for change detection. In terms of latency, the peak latencies of cortical On/Off responses, cortical OSRs, and the MMN all fall in the range of 100–200 ms [2, 63, 76, 79, 112]. In terms of spatial distribution, the sources of cortical Off response and MMN are similar. As revealed in animal studies, the sources of Off responses appear to be in the non-tonotopic area adjacent to the tonotopic area [4, 97]. In dense mapping MMN studies, the pitch MMN was reported to be generated in the secondary auditory area (or spreading more widely over the core and belt areas). This is distinct from the sources of the P1 and N1, at the core areas (A1 and AAF) [73, 92]. Cortical responses to the onset, offset, and pitch change of a continuous stimulus all share similar topography and temporal profiles, as suggested in several EEG/MEG studies [68, 115, 116]. Deviance-related responses also show similarities in their dependency on several factors regarding the regularities (e.g., probability of deviant, randomness in SOA, number of repetitions, effect of the NMDA-r antagonists) and the deviance magnitude (e.g., the sharpness in temporal, spectral, contextual changes). These observations support the notion of a common neural substrate of change detection for different deviance-related responses.

*The recurrent nature of the intracortical wiring makes change detection ubiquitous.* Functionally speaking, the ubiquity of change detection across the brain facilitates perceptual representation across the hierarchy. Edge information at all levels, provided by the local change detectors, augments the representational space. Such information compression may also contribute to energy saving. In this sense, the change detectors are more like high-pass filters than comparators that subtract top-down signals from the bottom-up signals. The abundant recurrent wiring patterns in the cortex provide a suitable environment for the emergence of change detectors. We take the diversity of cortical On/Off responses [18, 24, 106] as an example. Even though these responses could originate from the feed-forward mixture of non-cortical On/Off responses at earlier stages such as the thalamus, midbrain, and brainstem, the cortex provides more abundant chances for the emergence of On/Off responses. In simulation I, we demonstrated that various types of On/Off responses can be generated by different inter-node connections (Fig. 2). In simulation II, we further demonstrated that for a specific connection setting, the difference in input ratios to nodes gives rise to distinct onset and offset FRFs (Fig. 3). The *W* solutions of On/Off responses projected onto the 2D plane (Fig. 2c) also provided an explanation for the diverse (and spatially clustered) cell responses observed in auditory cortex in awake mice, as shown in Figure 5 in [24]. These results suggest that change detection is a basic and ubiquitous operation in the cortex.

We then study the generation of On and Off responses. On responses were due to a transient disinhibition (i.e., a quick and light inhibition on the inhibitory population of the change detector) before the network reached the steady state (Fig. 6). Off responses were always associated with a release from long-lasting disinhibition (i.e., a long and strong inhibition on the inhibitory population of the change detector) before the network came back to the steady state without the stimulus (Fig. 7). This is in line with the rebound after inhibition hypothesis [31, 97]. We suggest that the inhibitory-to-inhibitory connections are a key aspect of change detection.

*NMDA-r antagonists dampen the deviance-related responses.* We suggest that the NMDA-r antagonists could generally dampen the deviance-related responses through three aspects: (1) voltage dependency, (2) synaptic plasticity, and (3) *E*/*I* balance. First, the NMDA-r antagonists block the voltage-dependent NMDA channels and reduce the additional NMDA currents that reflect mismatch signals [41]. Second, the antagonists damage the spike-timing-dependent plasticity (STDP) and hamper the ability of regularity formation [4, 42, 103]. Third, the NMDA-r antagonists alter the connection patterns and *E*/*I* balance. Blocking NMDA receptors leads to decreased activity in the GABAergic interneurons and increased pyramidal excitation, because the GABAergic interneurons are tenfold more sensitive to the NMDA-r antagonists than the pyramidal neurons [27, 80].

The adaptation-based and prediction-based models of MMN agree on the voltage-dependency aspect and suggest that the reduced MMN amplitude is due to the reduction in NMDA currents [58, 107, 108]. The prediction-based models also mention the need for STDP to form prediction signals [107, 108]. In addition to these two aspects, our simulation results show that the altered *E*/*I* balance, as an effect of NMDA-r antagonists, can reduce the emergence of change detectors. In condition III (Fig. 8c), we reduced the strengths of $W^{EE}$ and $W^{IE}$ by 25$\%$ and 50$\%$, respectively, and recounted the number of each of the On/Off types in the scanned range of inter-node connection *W*s. The number of Off types decreased, whereas the number of On types is slightly increased relative to the default setting (Fig. 8h). These results suggest that the NMDA-r antagonists may dampen the cortical Off response, cortical OSR, and the MMN.

We cannot draw further quantitative conclusions from the effect of NMDA-r antagonists because the uniform search range of *W*s in the simulation is just a simplification. The exact proportion of strength reduction due to NMDA-r antagonists is not available. The settings of 25$\%$ and 50$\%$ in connection strength reduction in condition III were arbitrary so that a single node still oscillates under a certain range of input intensity, which eliminates the case when the nodes are saturated and no On/Off responses are generated at all. The time constant $\tau _e$, due to the blockage of NMDA channels, was not modified in the simulation in order to focus on the effect of *W* change.

*Synaptic adaptation facilitates change detection.* Synaptic adaptation is a pervasive short-term plasticity that is considered as a mechanism underlying deviance detection, in the sense that a rare stimulus triggers stronger neural activity via un-adapted pathways. Given the pervasiveness of synaptic adaptation, we were interested in how it affects the behavior of change detectors in our simulations. In condition IV (Fig. 8d), the strength of $W^{EE}$ was modulated by short-term adaptation according to the activity of pre-synaptic excitatory populations. After scanning through the *W*s, we found that the number of *W* solutions of both On and Off types was increased compared with the default condition (Fig. 8g). More specifically, many *W* solutions of None type turned into On and Off types when synaptic adaptation was applied (as examples in Fig. 9 show). We suggest that synaptic adaptation facilitates change detection by turning many otherwise None type responses (usually reflected by saturated activity in the excitatory populations) to either On or Off responses.

*The OSR is not just sustained resonance.* The OSR is differentiated from the Off response by its peak latencies that are proportional to the SOA in repetitive stimuli, reflecting the role of temporal expectancy. To maintain a short continuation of neural activity (i.e., sustained resonances) that preserves the periodicity of the repetitive stimuli, models that claim to account for the OSR utilize either an *adaptive approach* [99] or *population coding approach* [57]. However, sustained resonances alone cannot fulfill all observations in terms of peak amplitude and peak latency of the response. First, for the peak amplitude, the OSR cannot simply rely on the sustained resonance since the amplitude of OSR can be stronger than the evoked response during entrainment [33]. Second, for the peak latency, there should be a constant delay following the due time after stimulus offset [2, 90], but the sustained resonance rises exactly at the due time. Therefore, even though the sustained resonance is time-locked to the subsequent stimulus, there seems to be additional neural circuits responsible for the extra delay in peak latency and the stronger peak amplitude than the evoked responses. In simulation III, we demonstrated that the simulated OSR solves the two issues mentioned above (Fig. 4). Our model suggests that the cortical OSR can be interpreted as a cortical Off response at the end of sustained resonance. The simulation results are also in line with the finding that there is a pre-activated response at the time of expected onset followed by a mismatch response [2, 11, 85].

*The OSR is not a prediction signal.* The *omission paradigm* is often used to differentiate the contribution of *adaptation* and *prediction* in MMN generation. This is based on the assumption that the OSR could not arise without a stimulus and the involvement of active prediction. Interestingly, the models based on either the adaptation or prediction hypotheses interpret the OSR as essentially different from the MMN that is triggered by the classic oddball paradigm. In the adaptation-based model, the OSR is regarded as a rebound response (i.e., sustained resonance) rather than a delayed N1 [57, 58]. In the prediction-based model, the OSR is regarded as a pure prediction signal that originates from the memory unit rather than prediction error [108]. Both interpretations imply that the OSR is essentially different from the MMN because no additional NMDA current is generated. The problem is that neither the rebound response nor the prediction signal explains the two observations in terms of amplitude and latency mentioned above. As demonstrated in simulations III and IV (Figs. 4, 5), we suggest that the cortical OSR and MMN are essentially the same, both being the activity of change detectors.

The *cross-modal omission paradigm* is also used to emphasize the need for prediction. The brain can predict an upcoming event (e.g., a handclap sound) from the preceding events of another modality (e.g., a silent handclap video, or self-paced button press), and an OSR is triggered if an expected stimulus is omitted. In a motor-auditory (MA) paradigm, participants show OSRs when the sound, expected to be initiated by the self-paced button press, is omitted [85]. In a visual-auditory (VA) paradigm, an OSR is elicited by occasionally omitting the sound that accompanied a handclap video [95]. To date, cross-modal OSRs have not been considered by computational models. How does the generic deviance detection principle view the OSRs in these cross-modal paradigms that seem to be bound to an active predicting process? Here, we provide our viewpoint. First, the prediction is likely to be supported by the association between the cross-modal events (e.g., handclap video or button press, followed by a sound stimulus) that have to be paired or learned (e.g., by Hebbian learning) in advance via direct or indirect connections. The existence of association is reflected by the pre-activation at 40 to 80 ms in the auditory cortex elicited by a visual event [95] or by a motor event [85, 95]. In the MA paradigm, there is no pre-activation in the auditory cortex in the random condition where the button press is followed by a randomly selected sound and there is also no OSR thereafter [85]. This suggests that 48 trials are not enough to associate the button press to all 48 sound samples. Second, due to the pre-activation in the auditory cortex, the MA and VA paradigms can then be regarded as classic oddball paradigms where the standard is a ‘weak–strong’ sound pair and the deviant is a ‘weak–omission’ sound pair. In this sense, the cross-modal omission paradigm resembles an ‘intensity MMN’ or ‘duration MMN’ paradigm rather than an omission paradigm. This analogy explains why OSRs are elicited in the VA and MA conditions but not in auditory-only conditions (like a classic omission paradigm) [95]. More specifically, the SOAs (average 1155 ms) in the paradigm are above the temporal window of integration (TWI) for temporal features such as periodicity, but still within the TWI for identity features such as intensity and duration. The analogy can be verified if the VA and MA conditions fail to elicit ‘omission’ responses when the SOAs are larger than TWI for the identified features. Based on this analogy, the deviance detection that takes place in the auditory cortex stands alone from the process of association. This would explain why the pre-activation does not differ when the chance of sound omission is 50$\%$ verses 12$\%$, while the mismatch response following the pre-activation depends on the proportion of omission trials for both VA and MA conditions [95]. Association is less likely to be reduced by the 50$\%$ omissions, whereas deviance detection relies much more heavily on probability. Taken together, given the pre-activation via association and the analogy to the classic MMN paradigm, computational models that account for the classic MMN (e.g., either prediction-based or not) could potentially also account for the mismatch responses in cross-modal omission paradigms. From the viewpoint of generic deviance detection principle, the process of deviance detection (including regularity formation and change detection) takes place locally in the auditory cortex, even in the case of cross-modal VA and MA paradigms.

*Testable predictions.* In terms of the location of response, there are some testable predictions of our model. First, The cortical Off response, cortical OSR, and MMN should show similar laminar profiles, for example sink in layer 2/3 [41]. Second, inhibited activity of inhibitory interneurons near the location of the deviance response should be observed during stimulus presentation (regularity formation). Taking the pitch MMN as an example (assuming cortical area A has the best frequency (BF) of standard tone A, area B has the BF of deviant tone B, and area X is the location of MMN), the inhibitory interneurons in area X should be inhibited by tone A. In addition, area X can be a broader area (which may still include area B) that surrounds area A. In terms of the effect of NMDA-r antagonists, there are also several testable predictions of our model. First, the cortical OSR should be sensitive to the NMDA-r antagonists as are the other MMNs. Second, the amplitude of entrainment to periodic stimuli in omission paradigms should also be reduced by NMDA-r antagonists. Note: this prediction may have been partially supported by impaired delta entrainment in patients with schizophrenia [54].
